# Implementation of team-based learning in year 1 of a PBL based medical program: a pilot study

**DOI:** 10.1186/s12909-016-0550-3

**Published:** 2016-02-04

**Authors:** Annette Burgess, Tom Ayton, Craig Mellis

**Affiliations:** Education Office, Sydney Medical School, University of Sydney, Sydney, NSW 2006 Australia; Central Clinical School, Sydney Medical School, The University of Sydney, Sydney, NSW Australia

**Keywords:** Team-based learning, Problem based learning, Medical program

## Abstract

**Background:**

A traditional and effective form of teaching within medical education has been Problem Based Learning (PBL). However, this method of teaching is resource intensive, normally requiring one tutor for every ten students. Team-based learning (TBL) has gained recent popularity in medical education, and can be applied to large groups of up to 100 students. TBL makes use of the advantages of small group teaching and learning, but in contrast to PBL, does not need large numbers of teachers. This study sought to explore the efficacy of using TBL in place of PBL in Year 1 of a medical program.

**Methods:**

In Year 1 of the medical program, two iterations of TBL, with 20 students, were run following four iterations of PBL within the Cardiology teaching block. Student feedback following PBL and TBL was collected by questionnaire, using closed and open ended questions. Additionally, individual and team tests were held at the beginning of each TBL class, and results of each week were compared.

**Results:**

All students (*n* = 20) participated in the test in week 1, and 18/20 students participated in week 2. In total, 19/20 (95 %) of students completed the questionnaires regarding their PBL and TBL experiences. The use of small groups, the readiness assurance tests, immediate feedback from an expert clinician, as well as time efficiency were all aspects of the TBL experience that students found positive. The clinical problem-solving activity, however, was considered to be less effective with TBL. There was a significant improvement (*p* = 0.004) in students’ score from the week 1 assessment (median = 2) to the week 2 (median = 3.5) assessment. Interestingly, all teams but one (Team 1) achieved a lower score on their second week assessment than on their first. However, the lowest performing team in week 1 outperformed all other teams in week 2.

**Conclusion:**

Students favoured many aspects of the TBL process, particularly motivation to do the pre-reading, and better engagement in the process. Additionally, the application of TBL principles meant the sessions were not reliant upon a large teacher to student ratio. Students, however, highlighted the need for more time within TBL for clinical problem-solving.

## Background

Challenges within tertiary education include reductions in university funding, increasing student numbers and decreasing academic staff numbers. Additionally, within medicine, the teaching of medical students relies heavily on public hospital medical practitioners who also have large clinical demands [1]. Moreover, workforce data indicates that many medical practitioners and educators will retire over the next 20 years, leaving a shortage in medical teachers [2, 3]. An obvious resource saving measure would be to increase student class size. However, large group lectures are not conducive to effective learning experiences for students, particularly within medical education, where small group active learning is optimal. There is a need to explore how traditional educational modalities might be modified to fit changing resource and workforce dynamics, as well as best practice in education.

A traditional and effective form of teaching within medical education has been Problem Based Learning (PBL). However, this method of teaching is resource intensive, normally requiring one tutor for every ten students. Team-based learning (TBL) has gained recent popularity in medical education [4]. TBL offers “an active learning and small group instructional strategy that provides students with opportunities to apply conceptual knowledge through a sequence of activities that includes individual work, team-work and immediate feedback” [5]. In its traditional format, TBL remains highly structured, with core design elements, and specific steps [5]. TBL has the potential to engage students in learning, develop a deep understanding of concepts, develop a sense of responsibility towards their teammates, and improve course performance [6, 7]. Generally involving multiple ‘teams’ of five to seven students, TBL can be applied to large groups of up to 100 students. In short, TBL makes use of the advantages of small group teaching and learning, but in contrast to PBL, does not need large numbers of tutors.

Currently, Sydney Medical School offers a four year graduate entry medical program, with a problem-based learning curriculum. During Year 1 and Year 2 of the program, students attend weekly PBL tutorials on university campus. Our study aimed to explore the efficacy of TBL in Year 1 of this medical program in terms of students’ perception of their experience in both PBL and TBL, and student test performance in TBL.

## Methods

### Participants

Convenience sampling was used, with 20 Year 1 medical students from two PBL groups participating in the study.

### Structure and content of the TBL

Students completed four weeks of Cardiology PBLs in traditional PBL format with the same tutor (one student facilitated 1.5 h PBL, plus one tutor facilitated 1.5 h PBL per week). Following this, two iterations of PBL were converted to TBL format. The same two PBL groups of 10 students each were combined to form one TBL class, consisting of four teams of five students. The TBL sessions were 1.5 h each in duration, with one session held each week. The TBL format consisted of [5]:

1. Team formation
2. Assigned Pre-reading
3. Individual Test (IRAT)
4. Team test (TRAT) using Immediate Feedback Assessment Technique, with ‘scratch’ cards
5. Immediate feedback from the supervisor who went through each question, guided by test results and student needs.
6. Problem-solving activity

Within the TBL sessions, the intent was to spend approximately 30 min on Steps 3–5 (IRAT, TRAT and Immediate feedback), and approximately 60 min of STEP 6 (Problem-solving activity).

The specific cardiology cases used for the TBL problem solving activities were:

Week 1: Infant with Down Syndrome and congenital heart disease.

Week 2: Young adult with sudden cardiac arrest from prolonged QT interval.

### Student feedback

A questionnaire was distributed to all participants immediately following the first two iterations Cardiology PBLs, and then immediately following two iterations of Cardiology TBL sessions. The questionnaires included nine closed items, using a likert-scale of 1 to 5, with 1 being ‘strongly disagree’, and 5 being ‘strongly agree’. Additionally, two open-ended questions were included. The questionnaire was adapted from a validated questionnaire designed by Thompson and colleagues (2009), to measure the quality of team processes in medical education [8].

Quantitative data were analysed using descriptive statistics. Thematic analysis was used to code and categorise qualitative data into themes. Once data had been coded and categorised into themes, the data within each theme were quantified in order to measure thematic prevalence [9].

### Student assessment (Readiness assurance tests)

#### Individual Readiness Assurance Test (IRAT)

An Individual Readiness Assurance Test (IRAT) was held at the beginning of each class in week 1 and week 2 of TBL. This test was in Multiple Choice Question (Single Best Answer) format, and consisted of 10 questions, each with five options. These assessments were analysed using the Wilcoxon Signed Rank Test.

#### Team Readiness Assurance Test (TRAT)

In their allocated teams, students undertook the same test as the Team Readiness Assurance Test (TRAT). However, this test (TRAT) was administered on a scratch card, with students instructed to stop once they had guessed the correct answer. Each team completed one scratch card together as a team. Teams who answered the question correctly on the first attempt received a score of 4, and those who answered correctly on the fifth attempt scored zero. These scores were then summed across the items to obtain a total score ranging from 0 to 40.

Ethics approval was gained from the University of Sydney Human Research Ethics Committee.

## Results

### Student feedback

**Fig. 1:**
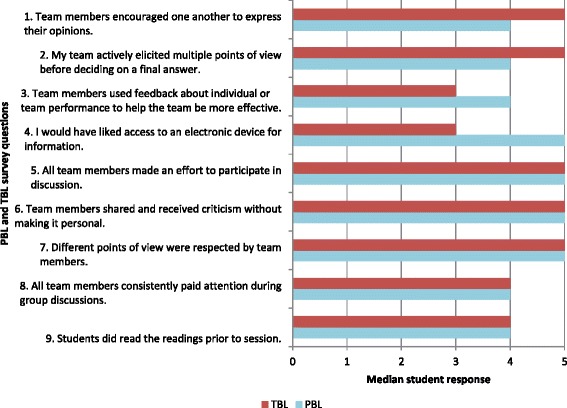
TBL features > PBL features: comparison of PBL and TBL, median student evaluation responses (*N* = 19)

In total, 19/20 (95 %) of students completed the questionnaires regarding their PBL and TBL experiences. The comparative results of these questionnaires are displayed in Fig. 1, which highlights students’ perceived advantages to TBL, and students’ perceived advantages to PBL. Table 1 displays the key performance statistics for PBL and TBL evaluation survey questionnaires.

**Table 1:** Key performance statistics for PBL and TBL evaluation survey questionnaires

Survey Question	PBL				TBL			
	n	Median	Standard Deviation	Mean	n	Median	Standard Deviation	Mean
Team members encouraged one another to express their opinions.	19	4	0.65	4.32	19	5	0.60	4.47
My team actively elicited multiple points of view before deciding on a final answer.	19	4	0.55	4.26	19	5	0.87	4.37
Team members used feedback about individual or team performance to help the team be more effective.	19	4	0.74	3.84	19	3	0.99	3.47
I would have liked access to an electronic device for information.	19	5	1.19	4.05	19	3	1.27	3.37
All team members made an effort to participate in discussion.	19	5	0.49	4.58	19	5	0.50	4.53
Team members shared and received criticism without making it personal.	19	5	0.44	4.74	19	5	0.75	4.42
Different points of view were respected by team members.	19	5	0.46	4.68	19	5	0.60	4.47
All team members consistently paid attention during group discussions.	19	4	0.57	4.32	19	4	0.59	4.42
Students did read the readings prior to session.	19	4	0.77	3.79	19	4	1.00	3.79

Table 2 illustrates students’ perceived best features of the PBL, and Table 3 illustrates students’ perceived worst features of the PBL. Table 4 illustrates students’ perceived best features of the TBL, and Table 5 illustrates students’ perceived worst features of the TBL.

**Table 2:** Student perceptions of the best features of PBL

Best features of PBL		
Summary	Student comment	No. of similar comments
Working through the clinical problem as a group	*“Working through the problem as a group, which provides multiple view points on various issues”*	12/19 (63 %)
Consolidating learning from lectures	*“Team works through a pattern specifically to consolidate learning from lectures”*	5/19 (26 %)

**Table 3:** Students’ perceptions of the worst features of PBL

Worst features of PBL		
Summary	Student comment	No. of similar comments
Time requirements for PBL lack efficiency	*“The sessions are very long, and it is sometimes challenging to keep everyone on track”*	10 (53 %)
Not everyone prepares for the PBL	*“Being prepared for PBL. Sometimes there are lots of readings. Hard to find time to be adequately prepared. Some people are not prepared”*	6 (32 %)
Information may not be correct	*“Varying opinions can sometimes lead to arguments about relevance, Not being sure of the validity of information from other group members”*	6 (32 %)
	*“Provision of tutors with relevant clinical experience to provide insight unattainable from normal study”*

**Table 4:** Students perceptions of the best features of TBL (*n* = 19)

Best features of TBL		
Summary	Student comments	No. of similar comments
The tests, both IRAT and TRAT motivated student to prepare, which made the session more engaging.	*“Team based tests created competition, and more motivation to prepare well”*	12 (63 %)
	*“Being forced to do pre-reading/pre-study made more feel more confident during the TBL session”*	
Small group size	*“The group discussion in the small groups (5) was very conducive to learning and overall participation in sessions”*	7 (37 %)
The presence of the facilitator and format of the session ensured accurate information, immediate feedback.	*“Having a knowledgeable tutor who could focus discussion was very valuable. …gave immediate feedback. We were able to have accurate information about the questions. In PLB we are unsure whether the information we provide is entirely correct”*	6 (32 %)
Time efficient	*“The TBL format was shorter than a PBL session, which was good because the PBL session felt too long for the content provided”*	6 (32 %)

**Table 5:** Students ‘perceptions of the worst features of TBL (*n* = 19)

Worst features of TBL		
Summary	Student comments	Number of similar comments
TBL lacked a clinical emphasis that is found in PBL	*“Unable to go through the clinical aspects of the case, management and mechanisms”*	12 (63 %)
There was not enough time allocated to the TBL session	*“Not having enough time allocated for the session meant the entire session felt a bit rushed”*	6 (32 %)
More specific pre-reading material was needed	*“Not enough specific readings assigned for preparation”*	6 (32 %)
The tests were difficult	*“Questions were very difficult, and isolated, rather than tied together with the case”*	3 (16 %)

### Student assessment (Readiness Assurance Tests)

#### Individual Readiness Assurance Test (IRAT)

20 students participated in the two week Team Based Learning (TBL) program. All students (100 %) participated in the individual 10-item assessment during Week 1 and 18/20 of these 20 (90 %) took the Week 2 assessment.

**Fig. 2:**
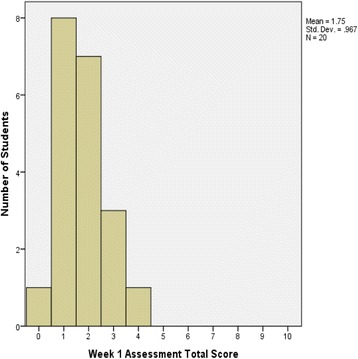
Total score distribution for TBL Week 1 IRAT assessment (*n* = 20)

**Fig. 3:**
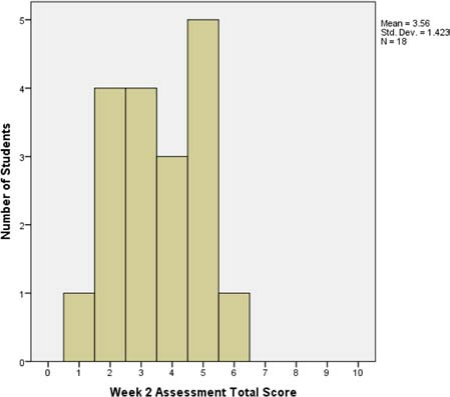
Total score distribution for TBL Week 2 IRAT assessment (*n* = 18)

Figures 2 and 3 show the difference between students’ total score on Week 1 and Week 2 assessments analysed using the Wilcoxon Signed Rank Test. There was a significant improvement (*p* = 0.004, *n* = 18) in students’ score from the Week 1 assessment (median = 2) to the Week 2 assessment (median = 3.5), with a median difference in score of 1.5. Twelve (12) students improved their scores from the 1st and 2nd week by 1 to 6 points. Four students showed no improvement, 3 with scores of 2 on both assessments, and one with a score of 3. Finally, two students scored lower on their 2nd week assessment than their first (by 2 and 1 points, for scores of 2 and 1, respectively).

#### Team Readiness Assurance Test (TRAT)

**Fig. 4:**
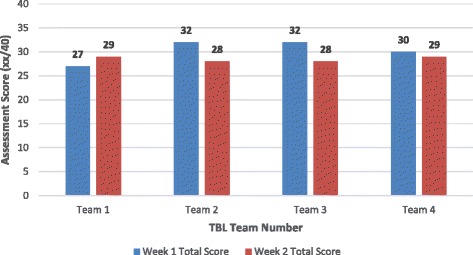
Comparison of TBL group assessment total scores by week

Interestingly, all teams but one (Team 1) achieved a lower score on their 2nd week assessment than on their 1st (Fig. 4). Teams 2 and 3 each scored 32 (80 %) on their 1st week assessment, the equal highest team scores, but both scored 28 (70 %) on their second week assessment. Team 4 scored the same each week: 30 (75 %) in 1st week assessment and 29 (73 %) on second. However, Team 1, managed to improve their score from 27 (67.5 %) on their 1^st^ week assessment to 29 (72.5 %) on their second week. Nevertheless, this team remained the lowest scoring team overall.

## Discussion

This study sought to explore students’ perceptions of their experience in both PBL and TBL. Students perceived both negative and positive elements to their experience of each educational modality. Students found working in smaller groups (of five) during the TBL more ‘conducive to learning’ and ‘participation’ than PBL (group size 10). Although they found the readiness assurance tests difficult, they also found the tests motivating and engaging. Students would have liked more specific reading material to assist in preparation. Students found immediate feedback from an expert tutor beneficial to their learning. The most notable negative aspect in TBL was it lacked the opportunity to work through the clinical aspects of a case. Although students found the TBLs to be ‘time efficient’, they commented that these sessions ‘felt a bit rushed’. Key elements of the TBL structure offer a useful framework to elaborate on these findings.

### Team dynamics

Reducing student numbers from ten (with PBL) to five (with TBL) appeared to be effective. Students commented that this enabled greater participation, collaboration and discussion. It has been suggested that student teams should be large enough to promote discussion yet small enough to maximise collaboration and team dynamics [10, 11]. Notably, responses to items 2 and 5 displayed in Fig. 1 indicate that team dynamics within TBL promoted greater student participation and discussion in TBL compared to PBL.

### Assigned pre-reading readiness assurance tests

In keeping with the current popularity of the ‘flipped classroom’, designated pre-class reading for essential knowledge acquisition for TBL shifts the burden of learning content during class [12]. Individual student accountability was fostered by the use of the individual assessment (IRAT), which also promoted effective teamwork. Students felt a sense of competitiveness among teams, which motivated them to come to class well prepared. A further indication of students’ motivation to prepare is that as individuals, most students improved their performance from week 1 to week 2 of the TBL. Notably, the poorest performing team (‘Team 1’) in week 1 was the best performing team in week 2. Perhaps this indicates that students from ‘Team 1’ felt challenged by their initial poor performance.

### Facilitator feedback

Students find it beneficial to receive immediate feedback from an expert tutor [13], and indicated that this is not always the case with PBL. Immediate feedback has the ability to provide students with an understanding of their content knowledge and ability to apply this [14]. Immediate feedback also contributed to the competition between teams. Provision of immediate feedback is well known to be crucial to knowledge acquisition and retention [15, 16].

### Problem-solving activity

Team learning in both PBL and TBL is promoted through implementation of the problem-solving activity [17]. During TBL, the small size of groups meant that all students were forced to contribute to the activity, increasing student engagement with the content. However, students noted that the teaching material and the lack of time meant that they were “unable to go through the clinical aspects of the case, management and mechanisms”. This issue is consistent with recent literature, suggesting that within health sciences education, the “problem-solving activity” step in TBL raises some difficulties. This difficulty with TBL needs careful consideration, and perhaps deviation from ‘classical’ TBL practice in medical education [18, 19].

### Limitations

A small sample size was used in this study, with only 20 students participating. It should also be noted that this was a pilot study, with only two iterations of TBL.

## Conclusion

The use of smaller groups, the readiness assurance tests, immediate feedback from an expert clinician, as well as time efficiency, were all aspects of the TBL experience that students found positive. Additionally, the application of TBL principles meant the sessions were not reliant upon a large teacher to student ratio. The clinical application to the problem-solving activity, however, appeared to be deficit in our experience with TBL. If TBL were to be implemented at Sydney Medical School in place of PBL, this step within the TBL process would need to be carefully designed.

## Abbreviations

TBL: Team-based learning
PBL: Problem based learning
IRAT: Individual Readiness Assurance Test
TRAT: Team Readiness Assurance Test
